# Towards Mimicking the Fetal Liver Niche: The Influence of Elasticity and Oxygen Tension on Hematopoietic Stem/Progenitor Cells Cultured in 3D Fibrin Hydrogels

**DOI:** 10.3390/ijms21176367

**Published:** 2020-09-02

**Authors:** Christian Garcia-Abrego, Samantha Zaunz, Burak Toprakhisar, Ramesh Subramani, Olivier Deschaume, Stijn Jooken, Manmohan Bajaj, Herman Ramon, Catherine Verfaillie, Carmen Bartic, Jennifer Patterson

**Affiliations:** 1 Department of Materials Engineering, KU Leuven, 3001 Leuven, Belgium; garciaabrego.christianjose@kuleuven.be (C.G.-A.); burak.toprakhisar@kuleuven.be (B.T.); 2 Department of Physics and Astronomy, KU Leuven, 3001 Leuven, Belgium; olivier.deschaume@kuleuven.be (O.D.); stijn.jooken@kuleuven.be (S.J.); carmen.bartic@kuleuven.be (C.B.); 3 Stem Cell Institute, KU Leuven, 3000 Leuven, Belgium; samantha.zaunz@kuleuven.be (S.Z.); bajajmsn@gmail.com (M.B.); catherine.verfaillie@kuleuven.be (C.V.); 4 Department of Biosystems, KU Leuven, 3001 Leuven, Belgium; ramesh.subramani@psgrkcw.ac.in (R.S.); herman.ramon@kuleuven.be (H.R.); 5 Department of Food Processing Technology and Management, PSGR Krishnammal College for Women, Coimbatore 641004, India; 6 IMDEA Materials Institute, 28906 Madrid, Spain

**Keywords:** fibrin hydrogel, elastic modulus, hematopoietic stem cells, fetal liver niche, 3D cell encapsulation, oxygen tension

## Abstract

Hematopoietic stem/progenitor cells (HSPCs) are responsible for the generation of blood cells throughout life. It is believed that, in addition to soluble cytokines and niche cells, biophysical cues like elasticity and oxygen tension are responsible for the orchestration of stem cell fate. Although several studies have examined the effects of bone marrow (BM) niche elasticity on HSPC behavior, no study has yet investigated the effects of the elasticity of other niche sites like the fetal liver (FL), where HSPCs expand more extensively. In this study, we evaluated the effect of matrix stiffness values similar to those of the FL on BM-derived HSPC expansion. We first characterized the elastic modulus of murine FL tissue at embryonic day E14.5. Fibrin hydrogels with similar stiffness values as the FL (soft hydrogels) were compared with stiffer fibrin hydrogels (hard hydrogels) and with suspension culture. We evaluated the expansion of total nucleated cells (TNCs), Lin^−^/cKit^+^ cells, HSPCs (Lin^−^/Sca^+^/cKit^+^ (LSK) cells), and hematopoietic stem cells (HSCs: LSK- Signaling Lymphocyte Activated Molecule (LSK-SLAM) cells) when cultured in 5% O_2_ (hypoxia) or in normoxia. After 10 days, there was a significant expansion of TNCs and LSK cells in all culture conditions at both levels of oxygen tension. LSK cells expanded more in suspension culture than in both fibrin hydrogels, whereas TNCs expanded more in suspension culture and in soft hydrogels than in hard hydrogels, particularly in normoxia. The number of LSK-SLAM cells was maintained in suspension culture and in the soft hydrogels but not in the hard hydrogels. Our results indicate that both suspension culture and fibrin hydrogels allow for the expansion of HSPCs and more differentiated progeny whereas stiff environments may compromise LSK-SLAM cell expansion. This suggests that further research using softer hydrogels with stiffness values closer to the FL niche is warranted.

## 1. Introduction

Hematopoietic stem cells (HSCs) are responsible for generating the full complement of mature blood cells throughout the lifespan of the animal [1,2]. During mammalian development, HSCs emerge from multiple regions, including the yolk sac, aorta-gonad-mesonephros (AGM), and placenta [3]. At around E11.5 of murine development or E28 in humans [4,5,6], HSCs colonize the fetal liver (FL), a highly vascularized organ that serves as the niche site of hematopoiesis during embryogenesis [7,8]. HSCs interact with other niche cells such as hepatoblasts [9,10], endothelial cells [11,12], and mural cells [4,7] that produce growth factors and cytokines important for HSC expansion and differentiation, including among others, stem cell factor (SCF), FMS-related tyrosine kinase 3 ligand (FLT3-L), and insulin like growth factor 2 (IGF-2) [13,14,15]. In the FL, HSCs undergo massive expansion, increasing their initial number up to 38-fold around E12.5–16 in mice and 6–22 weeks postconception in humans [6,16]. Moreover, FL is the site for the proliferation of downstream lineage cells, also termed hematopoietic progenitor cells (HPCs) [17,18,19]. While HSCs have the capacity to self-renew and to generate all blood cells, HPCs proliferate to a lesser extent and can only generate a part of the hematopoietic compartment (e.g., lymphoid or myeloid sub-compartment) [20]. In mice, hematopoietic stem/progenitor cells (HSPCs) (containing both HSCs and HPCs) are identified by the surface markers Lin^−^/Sca^+^/cKit^+^ (LSK), while additional markers like the so-called Signaling Lymphocyte Activated Molecule (SLAM) markers (CD150^+^ and CD48^−^) are used to distinguish HSCs (LSK-SLAM cells) from HPCs [21]. Shortly before birth, HSCs migrate to the bone marrow (BM), serving as the main niche site for adult hematopoiesis [3,8]. One important difference between FL-derived HSCs (FL-HSCs) and BM-derived HSCs (BM-HSCs) is that FL-HSCs are characterized by a much higher self-renewal and repopulation ability compared to BM-HSCs [22]. In the FL, HSCs undergo many symmetrical cell divisions to create the pool of HSCs required for life. In the BM, HSCs remain in a quiescent state, and if they divide, they usually make asymmetric cell divisions giving rise to one HSC daughter cell to maintain the HSC pool and one HPC daughter cell to fuel downstream hematopoietic compartments [23,24,25].

Deciphering the intrinsic and extrinsic mechanisms underlying the differences in self-renewal behavior of HSCs in the FL and BM niches remains a major question in the field, which also impacts the development of clinically relevant methods allowing true expansion of BM-HSCs ex vivo. A number of recent studies have focused on mimicking the BM niche characteristics in vitro by creating 3D culture systems that incorporate, along with growth factors and cytokines, different biomaterials to provide a range of biophysical cues in an attempt to induce symmetric cell divisions of HSCs in vitro [26,27,28,29,30,31,32]. Biomaterial elasticity is currently recognized to affect the fate of HSPCs [33,34,35,36]. In 2010, Holst et al. reported that the proliferation of repopulating HSCs was enhanced when cells were cultured on top of soft tropoelastin-coated plates compared to cells cultured on top of control plates without tropoelastin [33]. Since then, other authors have investigated the effects of different combinations of biophysical and biochemical cues in 2D or 3D systems on HSPC viability and morphology [35], adhesion and migration [37], as well as proliferation and differentiation [36,38]. Worth noting, these studies have mostly investigated the effects of stiffness values in the range of the BM niche (0.5 to 40 kPa) [39,40], whereas the elastic properties of FL, the in vivo organ where HSC expansion is most pronounced [6], have not been explored.

In addition to a material’s modulus, other factors such as oxygen tension have been reported to influence HSC and HPC behavior. For instance, BM-HSCs were maintained to a greater degree when cultured under 1–5% O_2_ (hypoxia) as compared to in normoxia [41,42], and hypoxic niches limit the production of reactive oxygen species (ROS) [43]. The importance of hypoxic environments in 3D systems was highlighted by a recent study from Braham et al. In that study, a co-culture of endothelial and adipose cells self-assembled into networks that created artificial hypoxic environments inside of Matrigel hydrogels even when cultured in conventional incubators (5% CO_2_ and 20% O_2_) [29]. These artificially hypoxic environments helped to preserve the pool of CD34^+^/CD38^−^ HSPCs (the human equivalent of murine LSK cells) encapsulated within the hydrogel. The human equivalent of murine LSK-SLAM cells was not investigated. Although the effect of oxygen tension on the expansion of HSPCs and HSCs has been assessed in suspension cultures, we are not aware of studies testing the expansion of HSPCs and HSCs when cultured inside hydrogels under different levels of oxygen tension.

We hypothesized that hydrogels with elastic moduli closer to that of the FL niche, which is specialized in extensively expanding the HSPC populations in contrast to the BM niche, might be more favorable for adult BM-HSC expansion and maintenance in 3D culture in vitro. To test this, we measured the elastic modulus of murine FL tissue and designed 3D fibrin hydrogels that mimic as closely as possible the elasticity of the natural tissue. Fibrin hydrogels have been widely used in tissue engineering applications and stem cell research due to their bioactivity and biocompatibility. Published studies using fibrin hydrogels have been performed with HSPCs co-cultured with umbilical cord-derived MSCs on top of the fibrin hydrogels [32]. However, in the present work, we studied the expansion of HSPCs embedded in fibrin hydrogels in 3D. Hydrogels with two different elastic moduli and suspension culture were investigated for the expansion of BM-derived Lin^−^/cKit^+^ cells over a period of 10 days in culture under hypoxia and normoxia. Cell expansion was analyzed at the levels of total nucleated cells (TNCs), Lin^−^/cKit^+^ cells, HSPCs (LSK cells), and HSCs (LSK-SLAM cells).

## 2. Results

### 2.1. Characterization of the Elastic Properties of FL Tissue and Fibrin Hydrogels

**Figure 1:**
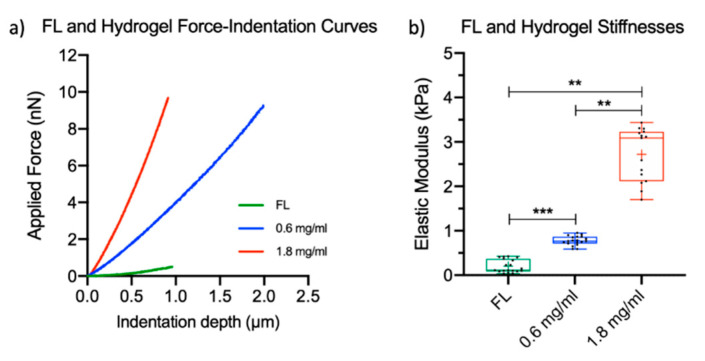
Elastic moduli of fetal liver (FL) tissue and fibrin hydrogels: (**a**) representative force-indentation curves of murine E14.5 FL tissue and fibrin hydrogels and (**b**) calculated Young’s moduli of FL tissue and fibrin hydrogels. Boxplots show the distribution of Young’s modulus with boxes representing the interquartile ranges, while the mean is represented by the plus sign. Dots represent the average values from the different areas. The mean elastic modulus of FL tissue was calculated using 19 randomly selected areas from 3 independent samples. The mean elastic modulus of the 0.6 mg/mL fibrin hydrogels was calculated from 19 randomly selected areas from 4 independent samples. For 1.8 mg/mL fibrin hydrogels, 15 randomly selected areas from 3 independent samples were used. Statistical analysis was performed with nested t-tests: ** *p* < 0.01 and *** *p* < 0.001.

Atomic force microscopy (AFM) was used to characterize the local elasticity of FL tissue and fibrin hydrogels under hydrated conditions. With this technique, mechanical properties such as Young’s modulus are measured by indenting the surface of the sample with a controlled force using a probe with a well-defined morphology (e.g., sphere, pyramid, and cone) placed at the end of a flexible silicon or silicon nitride cantilever. The indentation is given by the bending of the cantilever, measured with nanometer resolution by a laser beam reflected on the back of the cantilever falling onto a position-sensitive photodetector, while the applied force is precisely controlled by a piezoelectric scanner to which the cantilever is attached. The force versus indentation curves that are measured can be fitted with theoretical models to extract the static elastic modulus, as described in the Methods section. AFM is particularly interesting for mechanical property determination and mapping since it allows for probing the force and deformation with pN and nm resolutions, respectively, in physiologically relevant conditions [44]. Figure 1a shows representative force-indentation curves from FL tissue and fibrin hydrogels. The local elastic modulus values of murine FL tissue at E14.5 varied from 0.03 to 0.43 kPa with a mean value (± S.D.) of 0.20 ± 0.15 kPa (Figure 1b). For fibrin hydrogels, the elastic modulus values were 0.78 ± 0.10 kPa for hydrogels prepared with 0.6 mg/mL fibrinogen, referred as “soft” hydrogels, and 2.72 ± 0.60 kPa for hydrogels prepared with 1.8 mg/mL fibrinogen, referred as “hard” hydrogels (Figure 1b). The soft fibrin hydrogels had the closest modulus to murine FL tissue, although they were slightly stiffer. Hydrogels produced with concentrations of fibrinogen lower than 0.6 mg/mL were too fragile to be handled and therefore could not be used in this study.

### 2.2. Expansion of TNCs and of Lin^−^/cKit^+^, LSK, and LSK-SLAM Cell Populations

**Figure 2:**
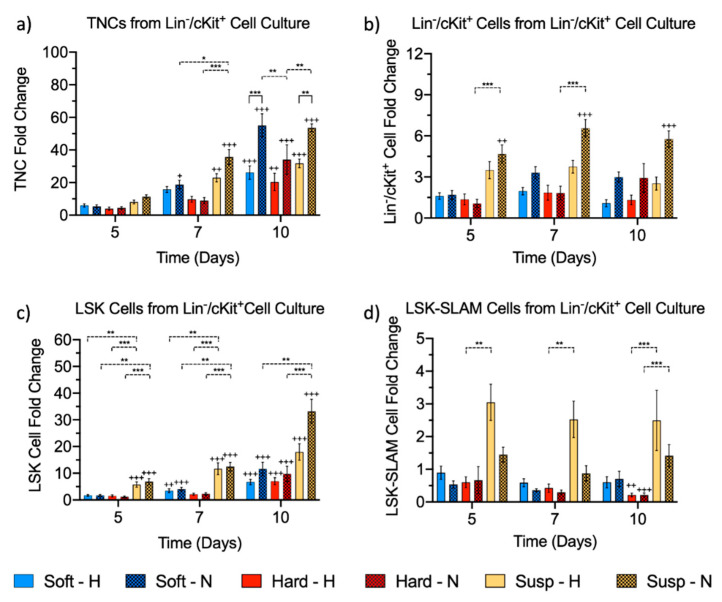
Quantification of total nucleated cells (TNCs) by NucleoCounter^®^ and hematopoietic stem/progenitor cell (HSPC) subgroups by flow cytometry retrieved from hydrogels (soft and hard) and suspension cultures (Susp) under hypoxia (H) and normoxia (N) relative to initial cell number over a culturing period of 10 days, shown as fold change of (**a**) TNCs, (**b**) Lin^−^/cKit^+^ cells, (**c**) Lin^−^/Sca^+^/cKit^+^ (LSK) cells, and (**d**) LSK-Signaling Lymphocyte Activated Molecule (SLAM) cells: data were obtained from 6 biological replicates in 3 independent experiments except for suspension cultures in hypoxia, which contained 4 biological replicates. Data are presented as mean ± S.E.M. Statistical analysis was performed with a three-way ANOVA followed by Tukey post hoc tests for multiple comparisons. Asterisks denote significance between the different culture conditions (soft hydrogels, hard hydrogels, and suspension culture) or between hypoxia and normoxia: * *p* < 0.05, ** *p* < 0.01, and *** *p* < 0.001. The plus signs denote significance between data collected at different time points against day 0: ^+^ *p* < 0.05, ^++^ *p* < 0.01, and ^+++^ *p* < 0.001.

To test our hypothesis that an in vitro cell culture model mimicking FL niche elasticity instead of commonly reported approaches mimicking BM niche elasticity might allow for expansion and maintenance of adult HSPCs ex vivo, Lin^−^/cKit^+^ cells were isolated from murine BM and embedded in soft and hard fibrin hydrogels or grown in suspension in wells of standard culture plates (control). All conditions were cultured for 10 days under both hypoxia and normoxia. TNC expansion starting from the Lin^−^/cKit^+^ cell population was assessed on days 5, 7, and 10 using a NucleoCounter^®^, and the fold change is reported as mean ± S.E.M. All culture systems supported a significant expansion of TNCs after 10 days (Figure 2a). In hypoxia, the highest overall TNC expansion was observed in suspension culture (32 ± 2.6-fold), followed by soft hydrogels (26 ± 4.1-fold) and hard hydrogels (20 ± 5.3-fold), but these differences were not statistically significant. In normoxia, the greatest cell number was observed for soft hydrogels followed by suspension cultures, with a similar expansion of 55 ± 7.2-fold and 54 ± 2.4-fold, respectively. However, hard hydrogels displayed the lowest cell number (34 ± 9.1-fold expansion). This was a significant reduction in the expansion compared to soft hydrogels and suspension cultures (*p* < 0.01). TNC expansion was significantly higher in soft hydrogels and suspension culture maintained in normoxia compared to conditions maintained in hypoxia (*p* < 0.001 and *p* < 0.01, respectively), with almost twice the TNC number after 10 days of culture.

Expansion of Lin^−^/cKit^+^, LSK, and LSK-SLAM cells was analyzed by Fluorescence Activated Cell Sorting (FACS), and the fold change is reported as mean ± S.E.M. In hypoxic conditions, no significant changes in Lin^−^/cKit^+^ cells were observed for all 3 culture conditions at day 10 compared to day 0 (Figure 2b). Under normoxic conditions at day 10, among the 3 culture conditions, the highest expansion was measured for suspension culture (5.8 ± 0.6-fold) followed by soft and hard hydrogels (3.0 ± 0.4 and 2.9 ± 1.0-fold, respectively). A trend of higher expansion in normoxia compared to that in hypoxia was observed although these differences were not statistically significant. After 10 days of culture under hypoxic conditions, LSK cells expanded significantly, up to 18 ± 3.1-fold in suspension cultures with 6.7 ± 1.0 and 7.0 ± 1.3-fold expansion in soft and hard hydrogels, respectively, and no significant differences between the hydrogels were observed (Figure 2c). A similar significant increase in LSK cells was observed in all normoxic culture conditions. LSK cells expanded up to 33 ± 4.5-fold in suspension culture, followed by 12 ± 2.5 and 9.8 ± 2.8-fold increases in soft and hard hydrogels, respectively. Similar to the Lin^−^/cKit^+^ cells, a higher LSK cell number was observed for normoxia relative to hypoxia on day 10, but this difference was not statistically significant. For LSK-SLAM cells, a 2.5 ± 0.9-fold increase was observed in suspension cultures under hypoxia, with cell loss for both hard and soft hydrogels after 10 days, i.e., 40% average loss of the initial cell number in soft hydrogels and 79% average loss in hard hydrogels (Figure 2d). This reduction in LSK-SLAM cell number in the hard hydrogels was statistically significant compared to both day 0 and to cells in suspension culture. Under normoxic conditions, no significant changes in cell number were observed for soft hydrogel and suspension cultures after 10 days. Suspension cultures showed a 1.4 ± 0.3-fold increase in cell number, and for the soft hydrogels, a 29% cell loss was measured. A significant LSK-SLAM cell loss of 79% also occurred in hard hydrogels after 10 days in normoxia. When comparing the effects of oxygen tension after 10 days, no significant differences in cell number were observed between hypoxic and normoxic conditions for the 3 culture systems.

### 2.3. Cell Viability in Fibrin Hydrogels

**Figure 3:**
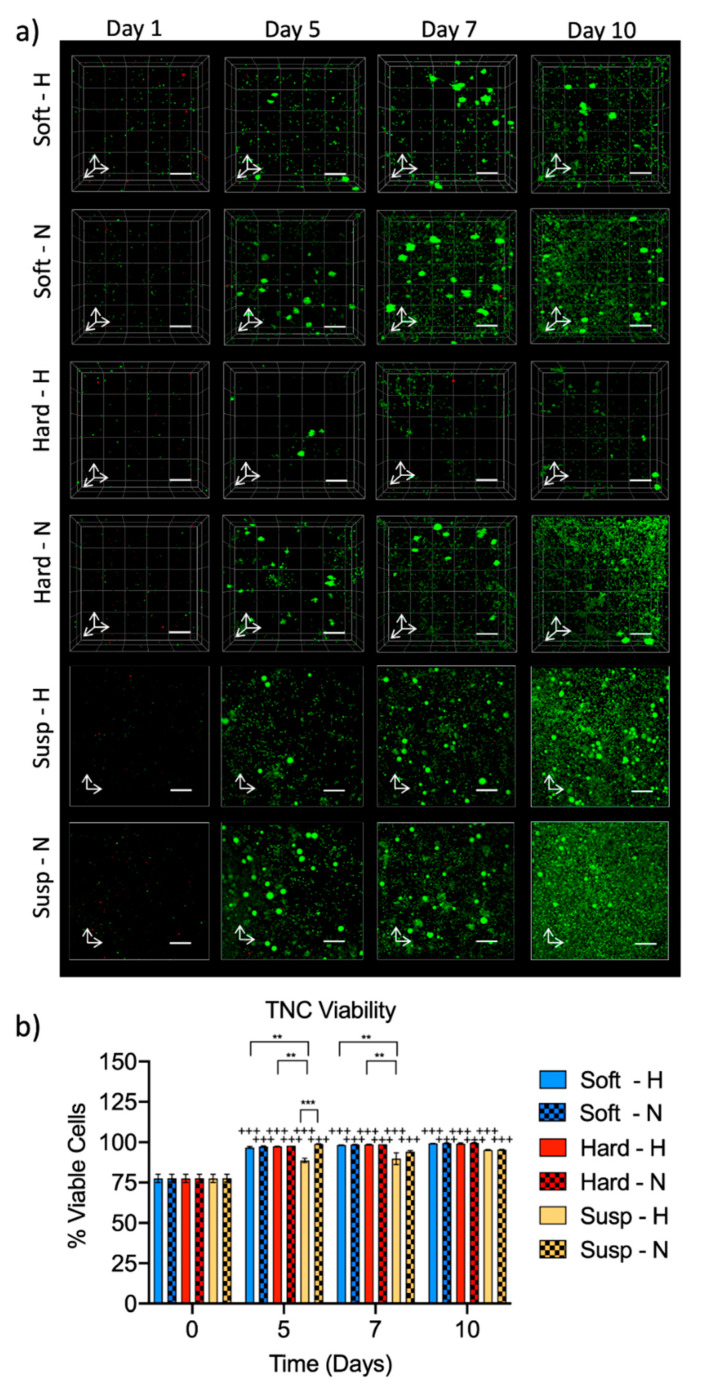
Fibrin hydrogels support the viability of Lin^−^/cKit^+^ cells: (**a**) confocal images of live (green) and dead (red) cells in soft and hard fibrin hydrogels as well as in suspension culture (Susp) grown in hypoxia (H) or normoxia (N) and (**b**) quantification of viability of Lin^−^/cKit^+^ progeny using the NucleoCounter^®^. Day 0 values are from the starting cell suspension. Scale bars are 200 μm, and white arrows indicate x- and y-axes for 2D images or x-, y-, and z-axes for 3D image stacks. Data were obtained from 2 biological replicates and are represented as mean ± S.E.M. Statistical analysis was performed with a three-way ANOVA followed by Tukey post hoc tests for multiple comparisons. The plus signs denote significance between data collected at different time points against day 0: ^+++^ *p* < 0.001. Other significant differences between groups are indicated with ** *p* < 0.01 and *** *p* < 0.001.

Cell viability was qualitatively assessed by confocal imaging using Live/Dead staining. We observed a homogenous distribution of cells in a fully 3D spatial confinement within the soft and hard hydrogels. After 10 days, cell expansion and high cell viability were observed for all culture conditions in both hypoxia and normoxia, with the formation of some clusters of cells in the hydrogels from day 5 onwards (Figure 3a). Confirmation that these larger, brightly stained regions in the Live/Dead images were formed by clusters of cells with a typical rounded morphology was performed by phalloidin and 4’,6-diamidino-2-phenylindole (DAPI) staining (Supplementary Figure S1). The cell viability was further quantified using the NucleoCounter^®^ (Figure 3b). Prior to encapsulation (day 0), the average viability of the starting cell suspension was 78%. Under both hypoxic and normoxic conditions and irrespective of the culture system, a significant increase in viability was measured by day 5 compared to day 0 (*p* < 0.001). After 7 and 10 days of culture, the viability remained ≥90% for all conditions.

### 2.4. LSK-SLAM Enriched Culture in Soft Hydrogels under Hypoxic Conditions

**Figure 4:**
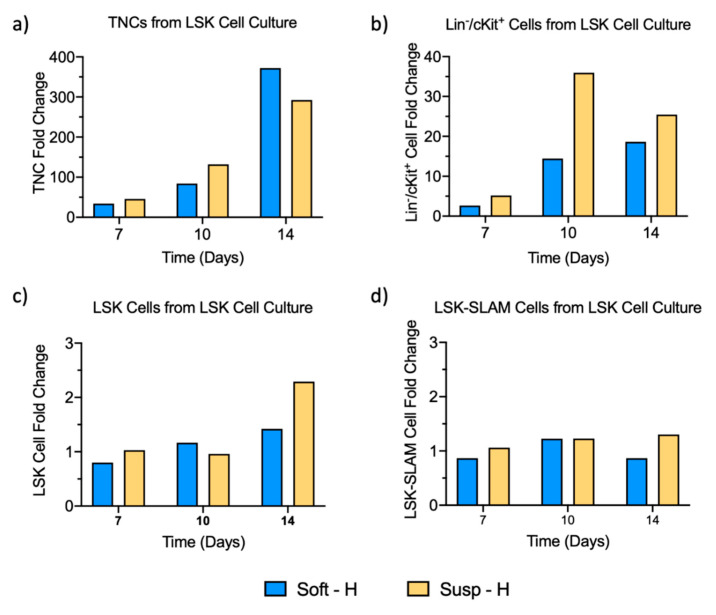
Quantification of HSPC subgroups from LSK-enriched cell culture, retrieved from soft fibrin hydrogels and suspension culture (Susp) under hypoxic conditions (H) over a period of 14 days, shown as fold changes of (**a**) TNCs, (**b**) Lin^−^/cKit^+^ cells, (**c**) LSK cells, and (**d**) LSK-SLAM cells: Data were acquired from 1 biological sample (pool of 6 donor mice). Given the rapid proliferation observed, cells were harvested on day 7, and the expanded cells were re-cultured under the initial cell conditions for another 7 days.

Given the low number of LSK-SLAM cells in the experiments starting with Lin^−^/cKit^+^ cells (mean ± S.D. = 12 ± 7 LSK-SLAM cells/sample), we also cultured FACS-sorted LSK cells, a more enriched LSK-SLAM population, to increase the number of HSCs. Because the number of LSK cells was much less than the number of Lin^−^/cKit^+^ cells harvested from mice, only 2 experimental conditions were selected based on those that best maintained the number of LSK-SLAM cells starting from Lin^−^/cKit^+^ cells, as shown in Figure 2d. LSK cells were embedded within soft fibrin hydrogels or grown in suspension culture without hydrogels as controls, with an average of 430 LSK-SLAM cells/sample, and cultured in hypoxia for 14 days. A rapid proliferation was observed for the LSK cell cultures, and thus, the expanded cells were harvested from hydrogels and suspension cultures on day 7, re-cultured at a low density, and expanded for another 7 days. After 14 days of culture, TNCs expanded 370-fold in soft hydrogels and 290-fold in suspension culture (Figure 4a). After 14 days, Lin^−^/cKit^+^ cells expanded 25- and 18-fold in suspension culture and soft hydrogel conditions, respectively (Figure 4b), whereas LSK cells expanded 2.3- and 1.4-fold in suspension culture and soft hydrogel conditions, respectively (Figure 4c). LSK-SLAM cells only exhibited a 1.3-fold change in suspension culture and a 0.9-fold change in the soft hydrogels after 14 days (Figure 4d). In general, starting from LSK cells led to the same trends as starting with Lin^−^/cKit^+^ cells, so using more purified cells was not explored further.

## 3. Discussion

In an attempt to mimic the mechanical properties of the FL niche, we prepared fibrin hydrogels with stiffness values comparable to and higher than murine FL tissue and evaluated their ability to support the expansion of murine BM-HSCs. To that end, we first performed AFM on FL tissue samples from E14.5 murine embryos. AFM is an excellent tool to determine the local elasticity of soft, heterogeneous materials, including biological tissue, with high spatial resolution in comparison to other techniques [44,45,46,47]. We initially tried to measure E12.5 FL, but this tissue was too soft and fragile, thus making slicing difficult. E14.5 FL tissue could be handled more easily and was also appropriate, as HSC expansion occurs in the FL between E12.5 and E16 [8]. For sectioning the tissue, an agarose gel was used according to an established methodology [48,49]. The measured FL modulus values were very low, with an average stiffness of 0.20 ± 0.15 kPa. The large standard deviation is likely due to the highly variable topography from the presence of extracellular matrix and various cell types and the viscous nature of the tissue.

To characterize the hydrogel moduli via AFM, hydrogels were tested in hydrated conditions. Our results indicate that the elasticity of the fibrin hydrogels increased with higher fibrinogen concentrations and that the variability around the mean increased with higher elasticities, which is consistent with previous reports [50]. Many parameters impact the elasticity of fibrin, making it difficult to directly compare our measurements with the results of previously published studies. For example, 20 mg/mL fibrin hydrogels had reported elastic modulus values of 9.29 to 15.78 kPa when prepared with different concentrations of NaCl, with higher concentrations leading to stiffer hydrogels [51]. These studies also used higher concentrations of thrombin (21–420 × compared to the recipe used herein) while factor XIII (FXIII) was not used [50,51]. Furthermore, variation in the concentration or ratio of thrombin and FXIII influences the elasticity of fibrin hydrogels. For example, a 1:1 ratio of thrombin–FXIII (the same as in our study) led to a 20-fold higher stiffness than a ratio of 1:0.1 when using the same fibrinogen concentration [52]. This is because the addition of FXIII is known to stiffen fibrin hydrogels through the compaction of fibrin fibers [53]. Moreover, the modulus values reported in different studies can only be compared when they are determined with the same technique, as the length scale of the mechanical probing has been shown to impact the values of the extracted parameters. With larger contact size, lower modulus values are generally determined [54,55]. In our case, the AFM characterization reflects the material mechanical properties at the micrometer scale.

To evaluate the influence of hydrogel stiffness on the viability and expansion of HSPCs, we encapsulated murine Lin^−^/cKit^+^ cells in soft and hard hydrogels (0.6 and 1.8 mg/mL fibrin hydrogels, respectively) or placed them in suspension culture for 10 days in hypoxic or normoxic conditions. The average moduli of the studied hydrogels were 0.78 kPa and 2.72 kPa, with the first closer to the modulus of the FL tissue (i.e., average modulus of 0.20 kPa), with all determined by AFM. We also attempted to prepare hydrogels with lower fibrinogen concentrations (e.g., 0.4 mg/mL); however, the fragility of these hydrogels rendered them impractical for cell encapsulation. Cells were precipitating at the bottom of the hydrogels during formation, and the hydrogels were losing integrity at the slightest manipulation, such as medium pipetting, so these very soft hydrogels were not used further. The fibrin hydrogels supported high cell viability, with ≥97% viable cells after 5 days, regardless of matrix elasticity or oxygen tension. This cell viability after several days of culture was much higher compared to previous studies encapsulating murine HSPCs in hydrogels. For example, a study by Cuchiara et al. showed only a viability of approximately 50% after 10 days in polyethylene glycol (PEG) hydrogels [27], whereas Mahadik et al. measured a viability of approximately 75% after 7 days in gelatin hydrogels [31].

Regarding cell expansion, TNCs expanded significantly in all culture systems after 10 days and hydrogel elasticity led to significant differences in TNC expansion in normoxia. Specifically, there was a greater expansion of TNCs in the soft hydrogels (0.78 kPa) compared to in the hard hydrogels (2.72 kPa). A similar TNC expansion behavior was reported by Bai et al. for cord blood-derived CD34^+^ cells encapsulated in zwitterionic hydrogels with a modulus of 0.70 kPa relative to cells in harder hydrogels (1.90 kPa), also cultured in normoxia [56]. Next, we evaluated the expansion of Lin^−^/cKit^+^ cells, HSPCs (LSK cells), and HSCs (LSK-SLAM cells) by flow cytometry. Although the highest expansion for all the hematopoietic populations was seen in suspension culture, both soft and hard fibrin hydrogels were able to significantly expand HSPCs over a period of 10 days. The hydrogel elasticity, in the investigated range, seemed not to affect the expansion of more committed progenitors, Lin^−^/cKit^+^ cells and LSK cells, as similar cell numbers were recovered from soft and hard hydrogels in hypoxic and normoxic conditions after 10 days. Our study also assessed the influence of hydrogel stiffness on HSC expansion and suggests that stiff environments may compromise LSK-SLAM cell expansion. However, softer hydrogels with a stiffness closer to the FL niche are better able to maintain LSK-SLAM cell number. Taken together, our data suggest that the HSPC expansion is less affected by hydrogel elasticity while HSCs may be negatively influenced by higher hydrogel stiffness.

Previous studies have also indicated that oxygen plays an important role in the expansion of HSPCs [41,43]. Thus, we investigated cell viability and expansion in both hypoxic and normoxic conditions. The TNC number was significantly higher in the soft hydrogels and in suspension culture when in normoxia compared to in hypoxia, and Lin^−^/cKit^+^ and LSK cells showed similar trends. On the other hand, the LSK-SLAM cell number remained similar in hypoxic conditions compared to in normoxic conditions over the 10 days of culture. Similar effects were also reported for HSPCs cultured in suspension in hypoxia or normoxia, suggesting that normoxia enhanced the expansion of TNCs and progenitors while hypoxia preserved FLT3^−^ CD34^−^ LSK cells (a subgroup of HSPCs between LSK and LSK-SLAM cells [57]) by inducing a quiescent state [42,58,59].

A key difference in our studies compared to previous work with fibrin and HSPCs is that the cells were encapsulated inside the hydrogels. In previous studies, HSPCs expanded more on top of substrates like fibrin hydrogels than in suspension cultures [32], but fibrin seems to have less of a positive effect on 3D-embedded cells, as observed in our study. Another study observed that, when HSPCs were cultured on top of substrates like PEG hydrogels, cell expansion was not affected by changes in the elasticity of the substrate. However, when those changes in elasticity were combined with spatial confinement (encapsulation), HSPCs responded with a decrease in cell cycling, being lower with higher stiffness and thus resulting in a lower expansion in stiffer hydrogels [36]. These results demonstrate once more that cell responses to biomaterials are very different in 2D and 3D conditions, given the differences in mechano-sensing and soluble biochemical factors [60]. Finally, none of the fibrin hydrogels were able to support HSC expansion. One possible explanation for why fibrin hydrogels failed to expand HSCs is the hydrophobic properties of fibrin [61]. Hydrophobic materials are known to trigger production of ROS molecules [62], which are also known to impede the self-renewal capabilities of HSCs [56]. A recently published study showed a 284-fold expansion of CD34^+^CD45RA^−^CD38^−^CD90^+^CD49f cells (the human equivalent of murine LSK-SLAM cells) when encapsulated in zwitterionic hydrogels with a stiffness similar to our soft system (0.7 kPa) [56]. To the best of our knowledge, this is the only study that has shown a true expansion of HSCs when encapsulated in 3D within hydrogels and not only maintenance of cell number, although they were working with cord blood-derived cells as well as an optimized cytokine cocktail in the medium. The capacity for substantial HSC expansion in zwitterionic hydrogels was attributed to the high hydrophilicity of these hydrogels, which helped to substantially reduce the production of ROS molecules [43].

## 4. Materials and Methods

### 4.1. FL Tissue Preparation and Elastic Modulus Quantification Via Atomic Force Microscopy (AFM)

FL tissues were obtained at E14.5 from C57Bl/6J mouse embryos. The use of animals and experimental procedures were approved by the Ethische Commissie Dierenwelzijn (ECD) of KU Leuven (project number 182/2016, approved April 2017). The isolated FL tissue was immersed immediately in a 1% agarose solution and maintained at less than 40 °C to avoid cell death in the tissue, prior to cooling down to form a gel. The FL-agarose gel was cut into 200-μm-thick slices using a microtome (Microm HM 360, Marshall Scientific, Hampton, NH, USA) at a speed of 15 μm/s. The tissue slices were immediately stored in phosphate buffered saline (PBS) (Gibco-Thermo Fisher, Waltham, MA, USA) at 37 °C and used for AFM measurements within 20–30 min. Prior to the AFM measurements, the slices were glued (JPK biocompatible glue) to a glass plate. Using a JPK NanoWizard III AFM, force–distance curves were recorded in force mapping mode at 37 °C by vertically indenting a colloidal AFM probe onto the tissue. The colloidal AFM probe consisted of a 20-µm diameter silica sphere attached to a triangular cantilever (PNPL probe, nominal spring constant k = 0.08 N/m, Nanoandmore GMBH, Wetzlar, Germany). The cantilever was calibrated using the thermal noise method implemented in JPKSPM software (version 5.0.135, JPK BioAFM, Berlin, Germany) before the measurements [63]. Nineteen randomly selected areas from 3 independent samples were measured at an indentation speed of 5 μm/s with a grid size of either 16 × 16 or 8 × 8 pixels. Young’s modulus (*E*) was calculated from the linear region of the force-indentation curves by fitting them with the Hertz contact model for a spherical indenter [64] using the JPK Data Processing Software (version 5.0.135, JPK BioAFM). For a spherical tip geometry, the relation between the force (*F*) and indentation (*δ*) is given by Equation (1):

(1) $$F=\left(\frac{4E\sqrt{R}}{3\left(1-\nu^{2}\right)}\right)\delta^{\frac{3}{2}}$$

with *E* = the elastic modulus, *R* = the tip radius, and *ν* = Poisson’s radio. A Poisson’s ratio of 0.5 was used to calculate E [65,66].

### 4.2. Fibrin Hydrogel Preparation and Determination of Elastic Modulus Using AFM

Fibrin hydrogels were prepared by mixing fibrinogen and thrombin components in equal volumes. The final concentrations used to prepare hydrogels with 0.6 mg/mL fibrinogen and 1.8 mg/mL fibrinogen are provided in Table 1. For the fibrinogen component, human plasminogen-depleted fibrinogen (Enzyme Research Laboratories, South Bend, IN, USA) was dissolved in a 20 mM 4-(2-hydroxyethyl)-1-piperazineethanesulfonic acid (HEPES) and 150 mM NaCl buffer (pH = 7.4), with or without the addition of cells. For the thrombin component, thrombin (Sigma, St. Louis, MO, USA), derived from human plasma, and human factor XIII (FXIII, Fibrogammin, CSL Behring, Mechelen, Belgium) were mixed in a 20 mM HEPES, 150 mM NaCl, 40 mM CaCl_2_, and 0.1% bovine serum albumin (BSA) buffer (pH = 7.4). The resulting thrombin-FXIII solution was incubated at 37 °C for 30 min in order to activate FXIII into FXIIIa. For AFM measurements, fibrinogen and thrombin components were then mixed to reach a total volume of 1000 μL and pipetted directly into a 35-mm petri dish to prepare samples with a thickness of about 2 mm to avoid underlying substrate effects. Subsequently, the hydrogels were left to polymerize for 30 min at 37 °C in a humid environment. Hydrogels were submerged into distilled water in order to keep them hydrated and were measured at room temperature (RT). The local stiffness of the fibrin hydrogels was assessed by AFM as described in Section 4.1, but a 10-μm diameter colloidal probe (PNPL probe, nominal spring constant k = 0.08 N/m, Nanoandmore GMBH) was used. For the 0.6 mg/mL fibrin hydrogels, 19 randomly selected areas (90 × 90 μm) from 4 independent samples were measured. For the 1.8 mg/mL fibrin hydrogels, 15 randomly selected areas from 3 independent samples were used. The areas were indented with a grid size of 8 × 8 pixels.

### 4.3. Isolation of Murine Lin^−^/cKit^+^ and Lin^−^/Sca^+^/cKit^+^ (LSK) Cell Populations

Lin^−^/cKit^+^ and LSK cells were isolated from bone marrow of 8–14-week-old C57Bl/6J mice. The use of animals and experimental procedures were approved by the ECD of KU Leuven (project number 182/2016, approved April 2017). Briefly, mice were euthanized by cervical dislocation in compliance with the KU Leuven Institutional Animal Care and Use Committee guidelines. Femurs and tibiae were collected, and cells were flushed out with PBS without calcium and magnesium. The cell suspension was centrifuged at 0.6 relative centrifugal force (RCF) for 7 min, after which erythrocytes were lysed using red blood cell lysis buffer (Biolegend, San Diego, CA, USA) for 10 min at RT. Cells were filtered through a 40-μm cell strainer (BD Falcon, Corning, NY, USA) to remove any tissue debris. Following filtration, cells were resuspended in Magnetic Activated Cell Sorting (MACS) buffer (PBS with 2 mM ethylenediaminetetraacetic acid (EDTA) and 0.5% BSA, Miltenyi Biotec, Cologne, Germany) at a concentration of 100E+6 cells/mL and incubated with a cocktail of biotin-conjugated anti-mouse lineage (Lin) antibodies (ABs) (CD3e, CD45R (B220), CD11b, Gr-1 (Ly-6G/C), and Ter-119 from eBioscience, San Diego, CA, USA) that was diluted 1/10 for 15 min on ice. Cells were then incubated for an additional 15–20 min with magnetic streptavidin beads (Biolegend, San Diego, CA, USA) that were diluted 1/40. The labelled cell suspension was then sorted via MACS columns (Miltenyi Biotec, Bergisch Gladbach, Germany). To obtain the Lin^−^/cKit^+^ cells, the negative fraction (Lin^−^) was collected and counted, and an additional cKit enrichment was performed using MACS CD117 microbeads (Miltenyi Biotec) according to the manufacturer’s protocol. Some Lin^−^/cKit^+^ cells were analyzed by flow cytometry to determine purity (see Section 4.7). The mean purity (±S.D.) of MACS-isolated Lin^−^/cKit^+^ cells was 68 ± 5.8%, with 86 ± 3.9% of cells being Lin^−^ cells, 2.7 ± 1.4% being LSK cells, and 0.22 ± 0.11% being LSK-SLAM cells. Prior to encapsulation, cell number and viability were quantified with a NucleoCounter^®^ NC-100^™^ using the propidium iodide (PI)-exclusion method according to the manufacturer’s protocol. The mean (±S.D.) cell viability before encapsulation was 79 ± 5.5% for the Lin^−^/cKit^+^ cells. Isolated Lin^−^/cKit^+^ cells were resuspended in StemSpan^TM^ Serum-Free Expansion Medium (SFEM; Stem Cell Technologies, Vancouver, BC, Canada) and kept on ice until further encapsulation or seeding in suspension culture.

For sorting of LSK cells, Lin^−^ cells were purified by MACS as described above from a pool of 6 donor mice, resuspended in FACS buffer (PBS with 0.4 mM EDTA and 0.1% BSA, Miltenyi Biotec) at 1 × 10^6^ cells/mL, and stained with fluorescently labelled ABs. The following ABs were used: fluorescein isothiocyanate (FITC) coupled anti-mouse Lin cocktail, allophycocyanin (APC) coupled anti-mouse cKit AB, phycoerythrin cyanine-7 (PECy7) coupled anti-mouse Sca-1 AB, phycoerythrin (PE) coupled anti-mouse CD150 AB, and allophycocyanin cyanine-7 (APC-Cy7) coupled anti-mouse CD48 AB. ABs were added diluted at 1/200, except PE CD150 AB was diluted at 1/100. All ABs except the anti-mouse Lin cocktail were from eBioscience. After 30 min, cells were washed and resuspended in FACS buffer. LSK cells were sorted by FACS (FACS Aria III cell sorter, BD Bioscience, San Jose, CA, USA). The compensation matrix was established using single-stained cells, and fluorescence minus one (FMO) controls were included to ensure proper gating and identification of the different cell populations of interest, as shown in Supplementary Figure S2. FACS-isolated LSK cells contained 7.7% LSK-SLAM cells. The mean cell viability before encapsulation determined using the NucleoCounter^®^ was 96% for the LSK cells.

### 4.4. Cell Encapsulation in Fibrin Hydrogels

Lin^−^/cKit^+^ cells were encapsulated in 0.6 mg/mL and 1.8 mg/mL fibrin hydrogels, whereas LSK cells were cultured only in 0.6 mg/mL hydrogels, with all at a final concentration of 200E+3 viable cells/mL. To prepare the cell-laden hydrogel samples, the fibrinogen and thrombin solutions (130 µL each) were prepared as described in Section 4.2 and then cast into 8 small droplets of 30 μL each, which were placed in separate wells of a 96-well plate (Nunclon™ Delta, Thermo Scientific). The polymerized hydrogels with encapsulated cells were submerged in SFEM medium supplemented with stem cell factor (SCF) and thrombopoietin (TPO) (Peprotech, Rocky Hill, NJ, USA) at 50 ng/mL each. The suspension cultures were prepared by culturing 6000 cells/well in 96-well plates (Nunclon™ Delta) under similar conditions as the hydrogels. Lin^−^/cKit^+^ cells were cultured for 10 days. LSK cells were cultured in hypoxia for 7 days, harvested and re-encapsulated in new hydrogels or re-seeded in suspension culture at the initial cell density, and cultured for another 7 days in hypoxia. Half of the medium was changed every 2 days throughout the experiments. During the encapsulation process of Lin^−^/cKit^+^ and LSK cells, 3 randomly selected fibrinogen-cell solutions per condition as well as 3 aliquots of cells in suspension were used for each biological replicate to quantify the actual number of TNCs at the start of the culture. To calculate the initial number of Lin^−^/cKit^+^ cells, LSK cells, and LSK-SLAM cells, the mean percentage of each subgroup obtained from FACS (Section 4.3) was multiplied by the TNC number for each culture condition. These initial cell numbers, considered as day 0, were used to calculate the fold change for each subgroup.

### 4.5. Cell Harvesting from Hydrogels and Suspension Cultures

Cells were harvested from the hydrogels by enzymatic digestion of the fibrin using nattokinase (NSK-SD; Japan Bio Science Laboratory, Osaka, Japan) dissolved at 50 fibrin degradation units per mL (FU/mL) in PBS containing 1 mM EDTA (Fisher Scientific, USA) [67]. Hydrogels were washed with PBS, and then, the nattokinase solution was added for the minimum amount of time to achieve hydrogel degradation, which was 15 and 30 min for the 0.6 and 1.8 mg/mL hydrogels, respectively. The nattokinase reaction was stopped by adding 200 μL of PBS with 10% fetal bovine serum (FBS). Cells were collected, centrifuged, and resuspended in FACS buffer. For suspension cultures, cells were collected directly without the addition of nattokinase.

### 4.6. Live/Dead Assays and Quantification of TNCs and Viability

Cell viability was analyzed 1, 5, 7, and 10 days after Lin^−^/cKit^+^ cell encapsulation. Cells in hydrogels and suspension culture were stained using a Live/Dead Viability Cytotoxicity Kit (Invitrogen, Carlsbad, CA, USA). Briefly, the medium was gently removed from the samples and replaced with PBS for 10 min. Thereafter, samples were stained with 2 μM calcein acetoxymethyl (AM) and 4 μM ethidium homodimer-1 in PBS at 37 °C. After 30 min of incubation, the dyes were removed and replaced with PBS prior to imaging. Cells at the center of the hydrogel and different areas of the suspension cultures were imaged using an inverted confocal microscope LSM 880 (Zeiss, Oberkochen, Germany) with a 10× objective lens. Z-stack images were collected for the cells in the hydrogels with a size of 1060 μm × 1060 μm × 350 μm. The interval between consecutive images in the stack was 5 μm. Calcein-positive (green) cells were viable, whereas ethidium homodimer-1-positive (red) cells were dead.

To quantify cell viability and TNC number, cells were harvested from the hydrogels or suspension cultures as described above. TNC counts were acquired using the NucleoCounter^®^ from 6 biological replicates from 3 independent experiments, except for suspension cultures in hypoxia, which contained only 4 biological replicates. Viability was quantified by PI-exclusion using the NucleoCounter^®^ from 2 biological replicates.

### 4.7. Flow Cytometry Staining and Data Acquisition

Flow cytometry was used to identify and quantify Lin^−^/cKit^+^, LSK, and LSK-SLAM markers prior to encapsulation and after culture within the hydrogels as well as in suspension. Cells were collected and resuspended in 100 μL FACS buffer, stained with fluorescently labelled AB cocktails on ice as described in Section 4.3, except for the anti-mouse CD48 AB, which was conjugated with eFluor450. All ABs were added diluted at 1/200. To quantify the different HSPCs present in each sample after culture via flow cytometry, counting beads (123count eBeads Counting Beads, Thermo Fisher Scientific) were added as an internal reference to each sample (10,090 beads per sample) prior to acquisition. This FACS-based cell counting method using the beads resulted in less intra-sample variation compared to a dual platform-based method, such as combining FACS data with the NucleoCounter^®^-based cell counts. However, the counting bead-based methodology consistently led to a lower average number of single cells per sample in all experimental groups compared to the numbers obtained using the NucleoCounter^®^ by about 70%, likely due to cell loss during the additional staining, washing, and centrifugation steps. Thus, the fold change values, which are relative to the day 0 values that were calculated based on the cell number from the NucleoCounter^®^, may be underestimated in all experimental conditions. Flow cytometry was performed using a BD Canto HTS II (BD Bioscience). To acquire enough events by FACS, 6 to 12 samples per culture condition were pooled at different time points. FACS data were analyzed with FlowJo software (version 10, FlowJo LLC, Ashland, Oregon, USA). A compensation matrix was established based on single-stained cells and FMO controls to ensure proper gating and identification of the different cell populations of interest (Supplementary Figure S3). For the Lin^−^/cKit^+^ cell encapsulation experiment, FACS data were collected from 6 biological replicates in 3 independent experiments, except for the suspension cultures in hypoxia, which contained only 4 biological replicates. For the LSK cell encapsulation experiment, FACS data were obtained using 1 biological replicate, pooled from 6 mice.

### 4.8. Statistical Analysis

Statistical analysis of cell counter and FACS data was performed using 3-way analysis of variance (ANOVA) in R (www.r-project.org) followed by Tukey-Honestly Significant Difference (HSD) post hoc tests for multiple comparisons. All fold changes calculated based on FACS data were log-2 transformed prior to statistical analysis. Nested t-tests were used to analyze the AFM data using GraphPad Prism software (version 8.3, San Diego, CA, USA). The significance level was set at α = 0.05.

## 5. Conclusions

We characterized the elasticity of murine FL tissue by AFM and found an average value of 0.20 kPa. Then, fibrin hydrogels were prepared to mimic as closely as possible the stiffness of the natural tissue. Soft (0.78 kPa) and hard (2.72 kPa) hydrogels were used to probe the effects of hydrogel elasticity on HSPC expansion in 3D systems, and these were compared with suspension culture in both hypoxia and normoxia. After 10 days, there was a significant expansion of TNCs and LSK cells in all culture conditions when compared to day 0, and normoxia supported to a greater extent the expansion of TNCs. LSK cells expanded more in suspension culture, and no significant differences were observed between the soft and hard hydrogels for this cell population. Furthermore, hydrogels with a stiffness similar to that of the FL tissue as well as suspension culture provided better maintenance of the number of LSK-SLAM cells in comparison to stiffer fibrin hydrogels. As next steps, it would be interesting to use materials varying in stiffness (including even softer hydrogels in the range of the FL stiffness) and hydrophilicity, combined with the co-culture of FL niche cells such as hepatoblasts, endothelial cells, and mural cells, to mimic the FL niche more accurately and to develop an in vitro model that supports HSC expansion for therapeutic applications.

## Figures and Tables

**Table 1:** Final concentrations for hydrogels with two different starting fibrinogen concentrations.

Component	0.6 mg/mL Fibrin Hydrogel	1.8 mg/mL Fibrin Hydrogel
Fibrinogen (mg/mL)	0.6	1.8
Thrombin (U/mL)	0.12	0.12
FXIII (U/mL)	0.12	0.12
CaCl_2_ (mM)	20	20
HEPES (mM)	20	20
NaCl (mM)	150	150
BSA (wt.%)	0.05	0.05

## Supplementary Materials

Supplementary materials can be found at https://www.mdpi.com/1422-0067/21/17/6367/s1. Figure S1: Confocal microscopy images showing HSPC morphology in fibrin hydrogels. Figure S2: FACS gates for the isolation of LSK cells. Figure S3: Flow cytometry plots to identify and quantify HSPC phenotype.

## Abbreviations

AB	Antibody
AGM	Aorta-Gonad-Mesonephros
AFM	Atomic Force Microscopy
AM	Acetoxymethyl
ANOVA	Analysis of Variance
APC	Allophycocyanin
APC-Cy7	Allophycocyanin Cyanine-7
BM	Bone Marrow
BM-HSC	Bone Marrow Derived Hematopoietic Stem Cell
BSA	Bovine Serum Albumin
DAPI	4’,6-Diamidino-2-phenylindole
E	Young’s Modulus
ECD	Ethische Commissie Dierenwelzijn
EDTA	Ethylenediaminetetraacetic Acid
FACS	Fluorescence Activated Cell Sorting
FBS	Fetal Bovine Serum
FITC	Fluorescein Isothiocyanate
FL	Fetal Liver
FL-HSC	Fetal Liver Derived Hematopoietic Stem Cell
FLT3-L	FMS-related Tyrosine Kinase 3 Ligand
FMO	Fluorescence Minus One
FU/ml	Fibrin Degradation Units per Milliliter
FXIII	Factor XIII
H	Hypoxia
HEPES	4-(2-Hydroxyethyl)-1-piperazineethanesulfonic Acid
HPC	Hematopoietic Progenitor Cell
HSC	Hematopoietic Stem Cell
HSD	Honestly Significant Difference
HSPC	Hematopoietic Stem/Progenitor Cell
IGF-2	Insulin like Growth Factor 2
Lin	Lineage
LSK	Lin^−^/Sca^+^/cKit^+^
MACS	Magnetic Activated Cell Sorting
N	Normoxia
PBS	Phosphate Buffered Saline
PE	Phycoerythrin
PECy7	Phycoerythrin Cyanine-7
PEG	Polyethylene Glycol
PI	Propidium Iodide
rcf	Relative Centrifugal Force
ROS	Reactive Oxygen Species
RT	Room Temperature
SCF	Stem Cell Factor
SFEM	Serum-Free Expansion Medium
SLAM	Signaling Lymphocyte Activated Molecules
Susp	Suspension
TNC	Total Nucleated Cell
TPO	Thrombopoietin
