# Supplementary material for: Towards Mimicking the Fetal Liver Niche: The Influence of Elasticity and Oxygen Tension on Hematopoietic Stem/Progenitor Cells Cultured in 3D Fibrin Hydrogels
Source: Int J Mol Sci. 2020 Sep 2;21(17):6367. doi: 10.3390/ijms21176367 (PMC7504340; doi:10.3390/ijms21176367)
Supplement: Supplementary file 1 [file ijms-21-06367-s001.pdf]

International Journal of *Molecular Sciences*

MDPI

Supporting Information

Towards Mimicking the Fetal Liver Niche: The Influence of Elasticity and Oxygen Tension on Hematopoietic Stem/Progenitor Cells Cultured in 3D Fibrin Hydrogels

Christian Garcia-Abrego [1,2,†], Samantha Zaunz [3,†], Burak Toprakhisar [1,3], Ramesh Subramani [4,5], Olivier Deschaume [2], Stijn Jooken [2], Manmohan Bajaj [3], Herman Ramon [4], Catherine Verfaillie [3], Carmen Bartic [2] and Jennifer Patterson [1,6,*]

1 Department of Materials Engineering, KU Leuven, 3001 Leuven, Belgium
2 Department of Physics and Astronomy, KU Leuven, 3001 Leuven, Belgium
3 Stem Cell Institute, KU Leuven, 3000 Leuven, Belgium
4 Department of Biosystems, KU Leuven, 3001 Leuven, Belgium
5 Department of Food Processing Technology and Management, PSGR Krishnammal College for Women, 641004 Coimbatore, India
6 IMDEA Materials Institute, 28906 Madrid, Spain
† Contributed equally
* Correspondence: pattersn@alumni.princeton.edu

Table of Contents:

Figure S1. Confocal microscopy images showing HSPC morphology in fibrin hydrogels p. 2

Figure S2. FACS gates for the isolation of LSK cells p. 3

Figure S3. Flow cytometry plots to identify and quantify HSPC phenotype p. 4-9

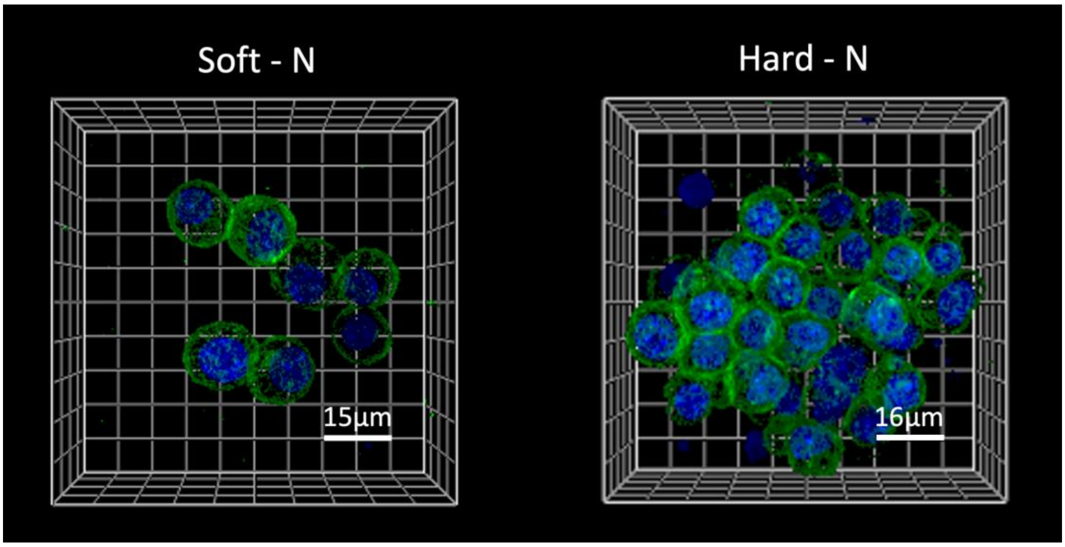

Figure S1. Confocal microscopy images showing hematopoietic stem/progenitor cell (HSPC) morphology in fibrin hydrogels. $Lin^-/cKit^+$ cells were encapsulated in soft (left image) and hard (right image) fibrin hydrogels and cultured in normoxia (N) for 10 days. Cells in the fibrin hydrogels were fixed, permeabilized, and stained with phalloidin and DAPI to show the morphology of the cytoskeleton (green) and nucleus (blue), respectively.

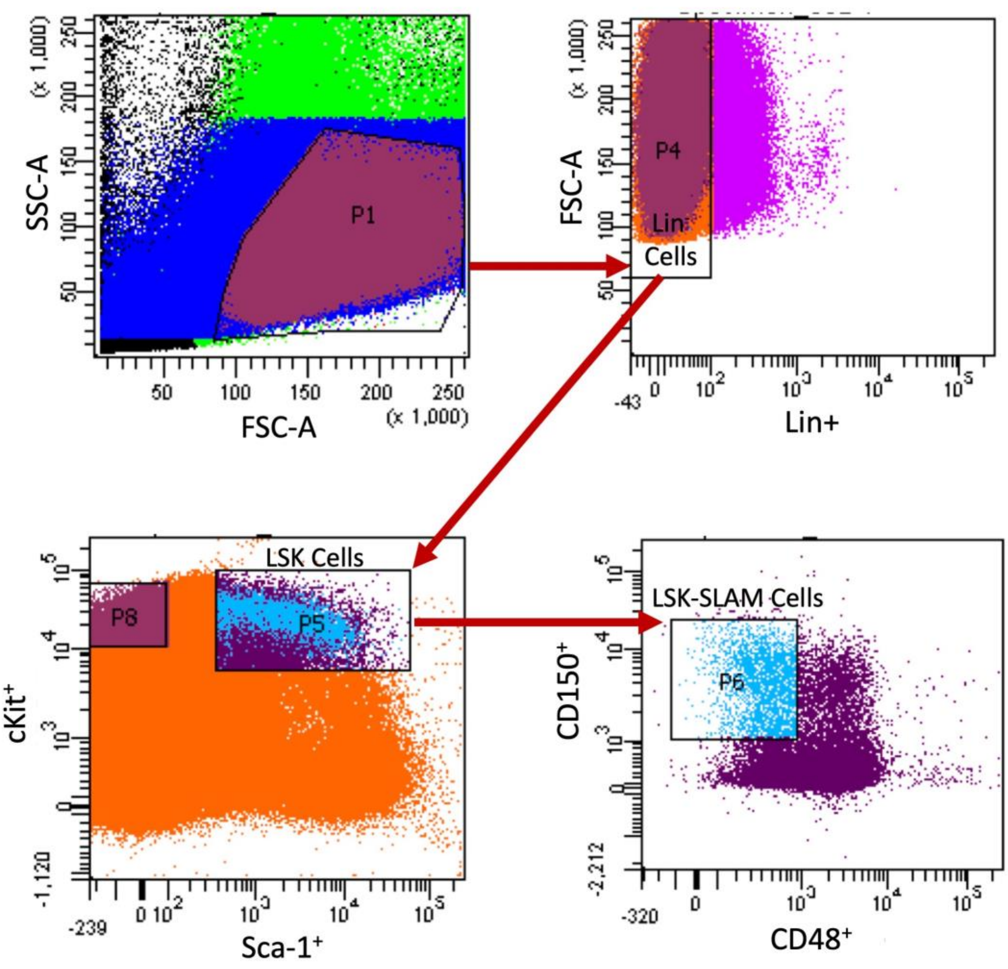

Figure S2. Fluorescence activated cell sorting (FACS) gates for the isolation of Lin$^-$/Sca$^+$/cKit$^+$ (LSK) cells from the bone marrow (BM) of femurs and tibiae of mice via flow cytometry. Freshly isolated BM cells were depleted of the lineage positive fraction by magnetic activated cell sorting (MACS). The lineage negative (Lin$^-$) fraction was collected and purified via FACS. P1 is the gate for the population of interest based on the forward and side scattering (FSC-A and SSC-A, respectively). P4 represents the gate for the Lin$^-$ population, followed by the P5 gate for LSK cells. Lastly, P6 denotes the gate for LSK-SLAM cells. P8 was not used and did not interfere with the sorting process. Gates were used from a custom-made template and compensated with the use of singly stained controls and fluorescence minus one (FMO) samples to properly gate and identify the cell population of interest using DIVA software (BD Biosciences). Approximately 200,000 LSK cells were obtained from 6 mice, with 7.7% being LSK-SLAM cells.

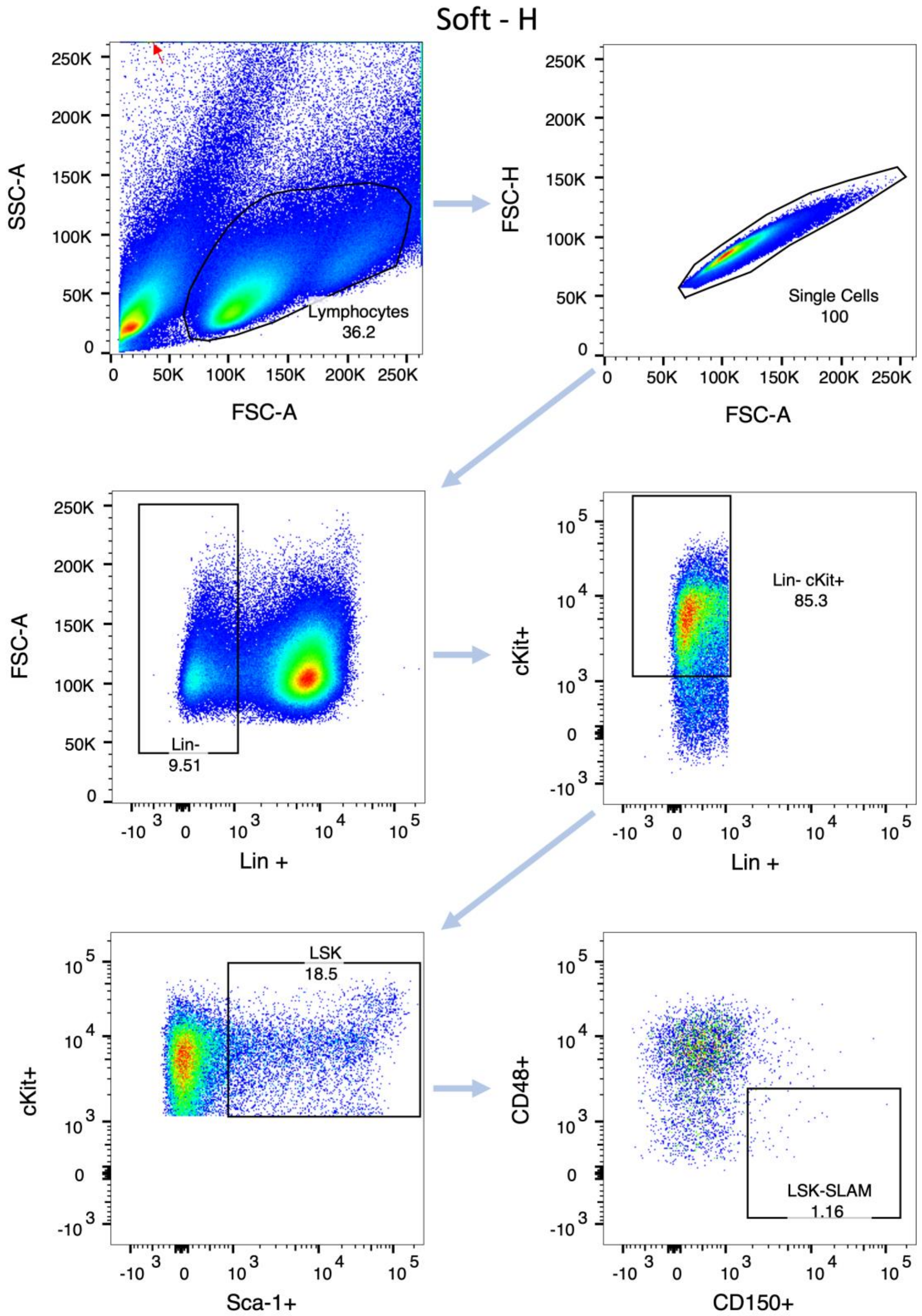

Figure S3a. Representative flow cytometry plots used to identify and quantify the hematopoietic stem/progenitor cell (HSPC) phenotype expression after 10 days of culture starting from Lin-/cKit+ cells within soft fibrin hydrogels under hypoxia (H). Small red arrows in the FSC-SSC plots denote the position of the counting beads. Debris and dead cells were excluded from the analysis by gating FSC-A/SSC-A. For a better discrimination, double gating with FSC-W/FSC-H and SSC-W/SSC-H was used (not shown).

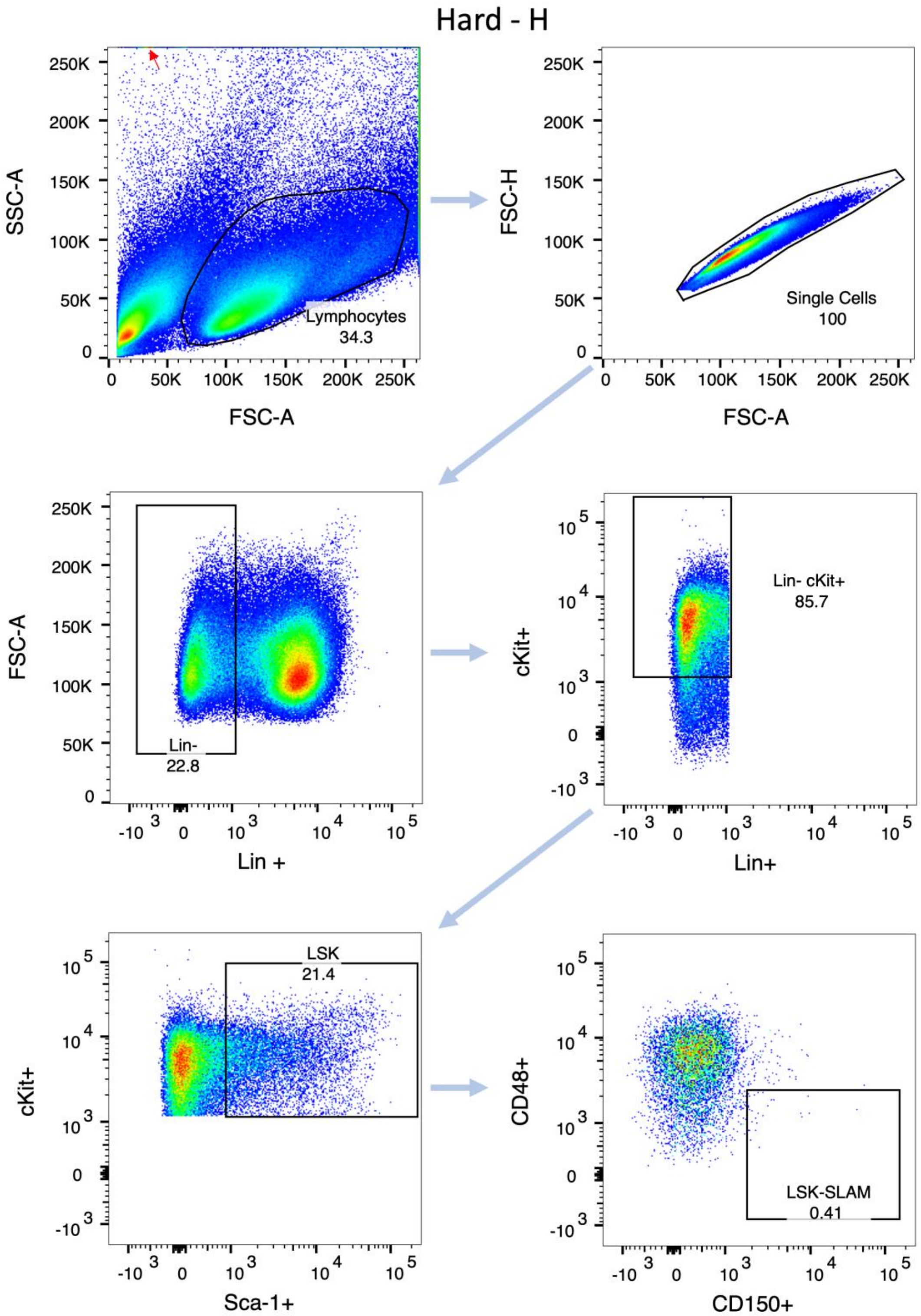

Figure S3b. Representative flow cytometry plots used to identify and quantify the hematopoietic stem/progenitor cell (HSPC) phenotype expression after 10 days of culture starting from Lin$^-$/cKit$^+$ cells within hard fibrin hydrogels under hypoxia (H). Small red arrows in the FSC-SSC plots denote the position of the counting beads. Debris and dead cells were excluded from the analysis by gating FSC-A/SSC-A. For a better discrimination, double gating with FSC-W/FSC-H and SSC-W/SSC-H was used (not shown).

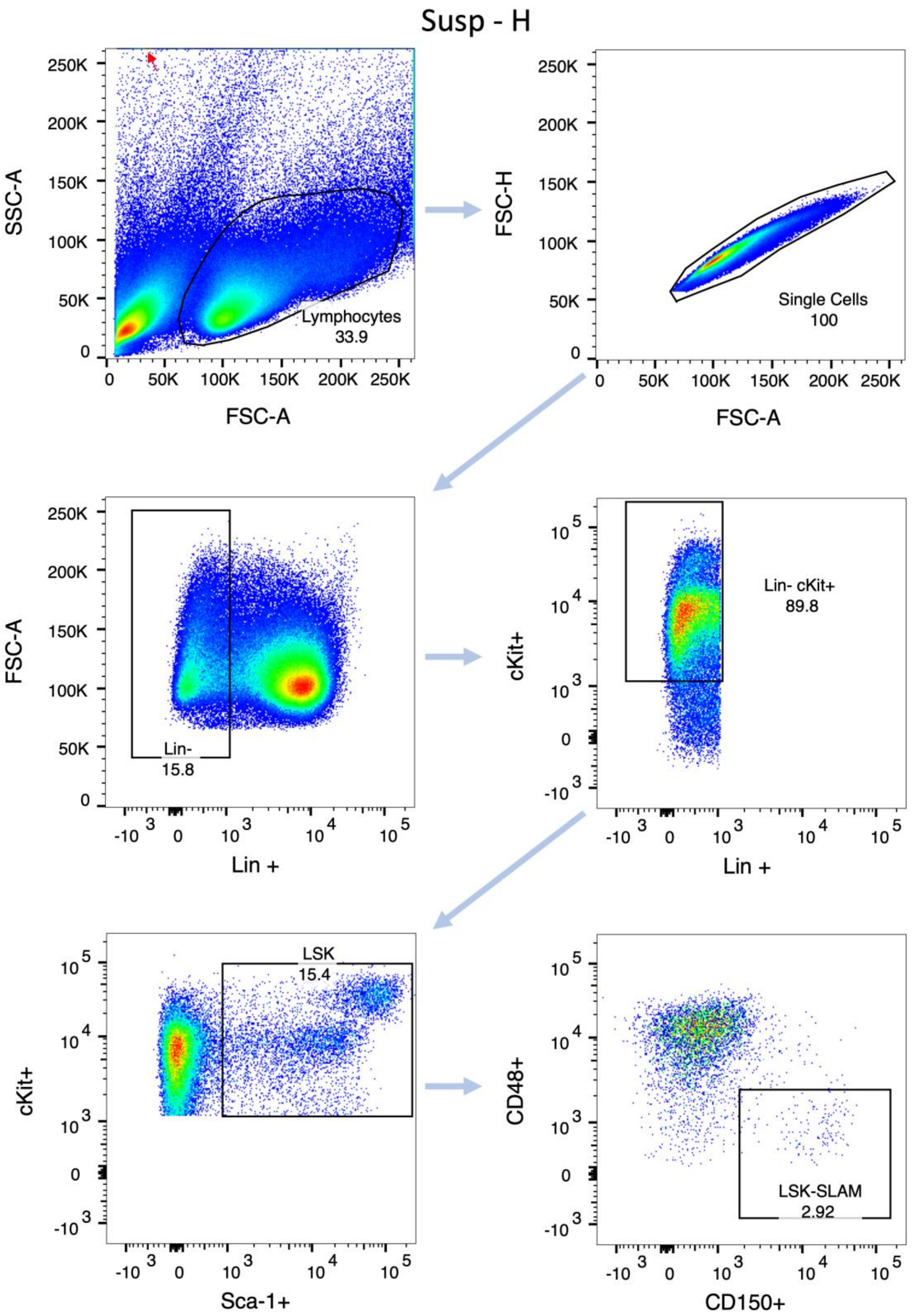

Figure S3c. Representative flow cytometry plots used to identify and quantify the hematopoietic stem/progenitor cell (HSPC) phenotype expression after 10 days of culture starting from $Lin^{-}/cKit^{+}$ cells in suspension culture (Susp) under hypoxia (H). Small red arrows in the FSC-SSC plots denote the position of the counting beads. Debris and dead cells were excluded from the analysis by gating FSC-A/SSC-A. For a better discrimination, double gating with FSC-W/FSC-H and SSC-W/SSC-H was used (not shown).

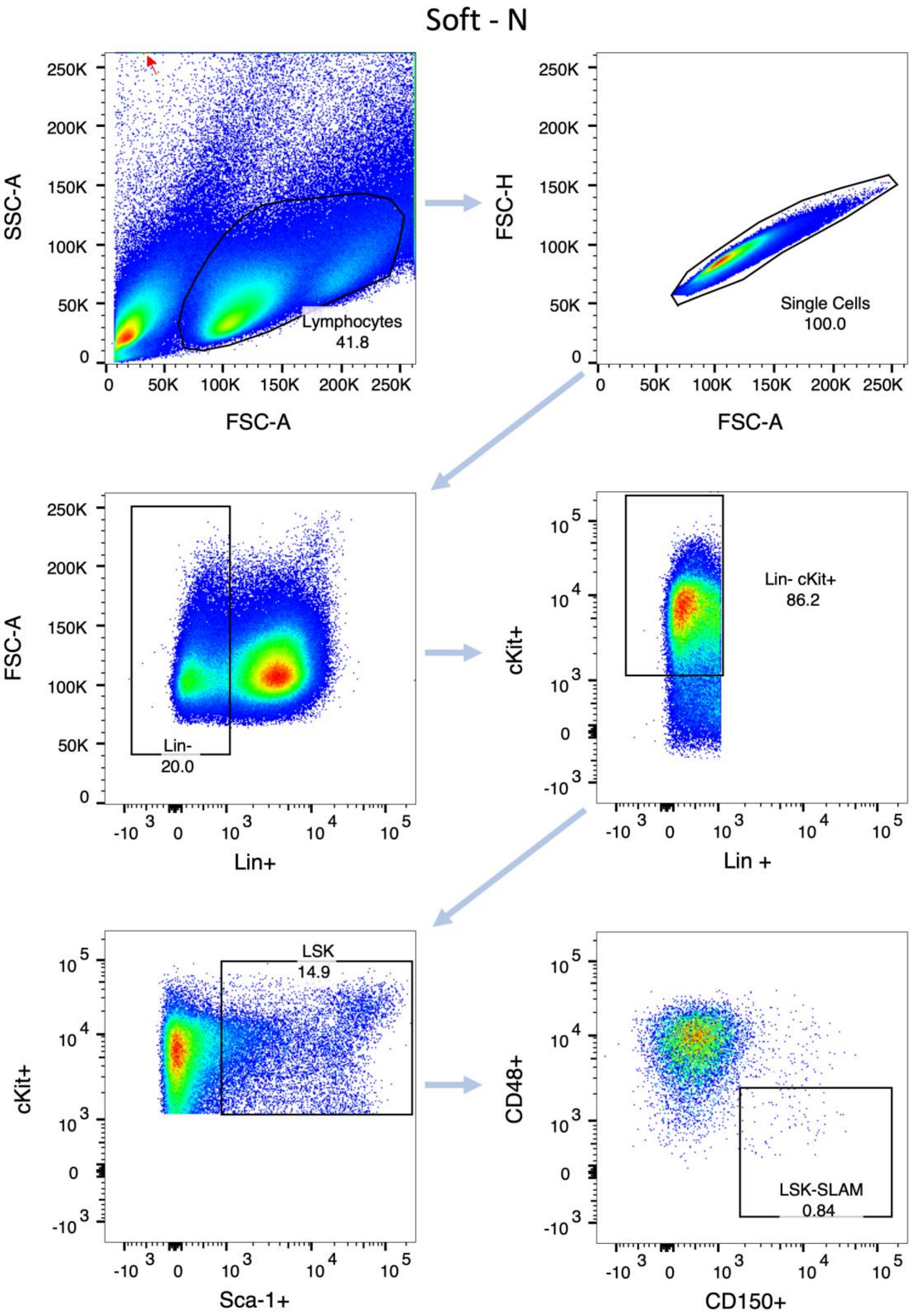

Figure S3d. Representative flow cytometry plots used to identify and quantify the hematopoietic stem/progenitor cell (HSPC) phenotype expression after 10 days of culture starting from Lin$^-$/cKit$^+$ cells within soft fibrin hydrogels under normoxia (N). Small red arrows in the FSC-SSC plots denote the position of the counting beads. Debris and dead cells were excluded from the analysis by gating FSC-A/SSC-A. For a better discrimination, double gating with FSC-W/FSC-H and SSC-W/SSC-H was used (not shown).

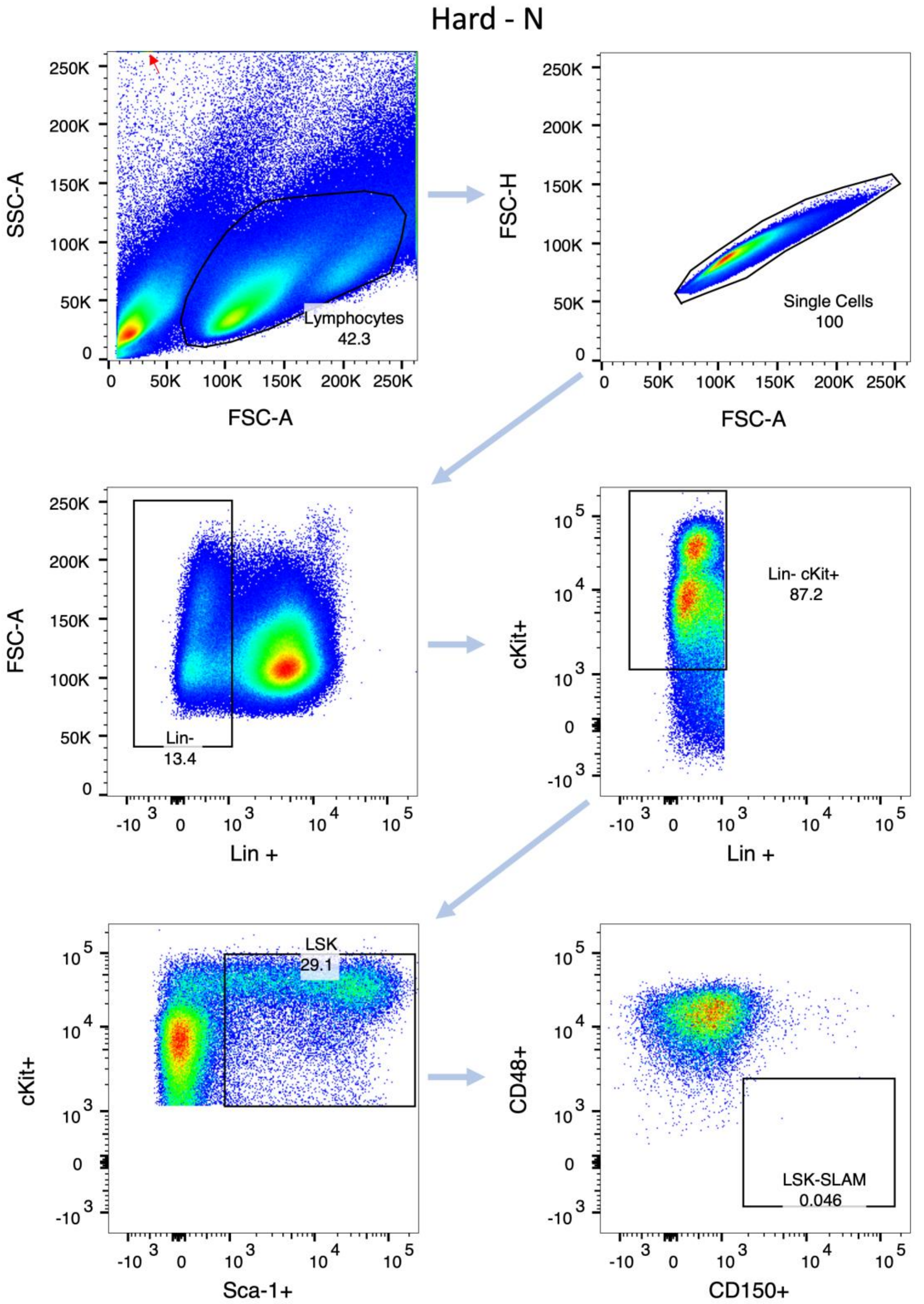

Figure S3e. Representative flow cytometry plots used to identify and quantify the hematopoietic stem/progenitor cell (HSPC) phenotype expression after 10 days of culture starting from Lin$^-$/cKit$^+$ cells within hard fibrin hydrogels under normoxia (N). Small red arrows in the FSC-SSC plots denote the position of the counting beads. Debris and dead cells were excluded from the analysis by gating FSC-A/SSC-A. For a better discrimination, double gating with FSC-W/FSC-H and SSC-W/SSC-H was used (not shown).

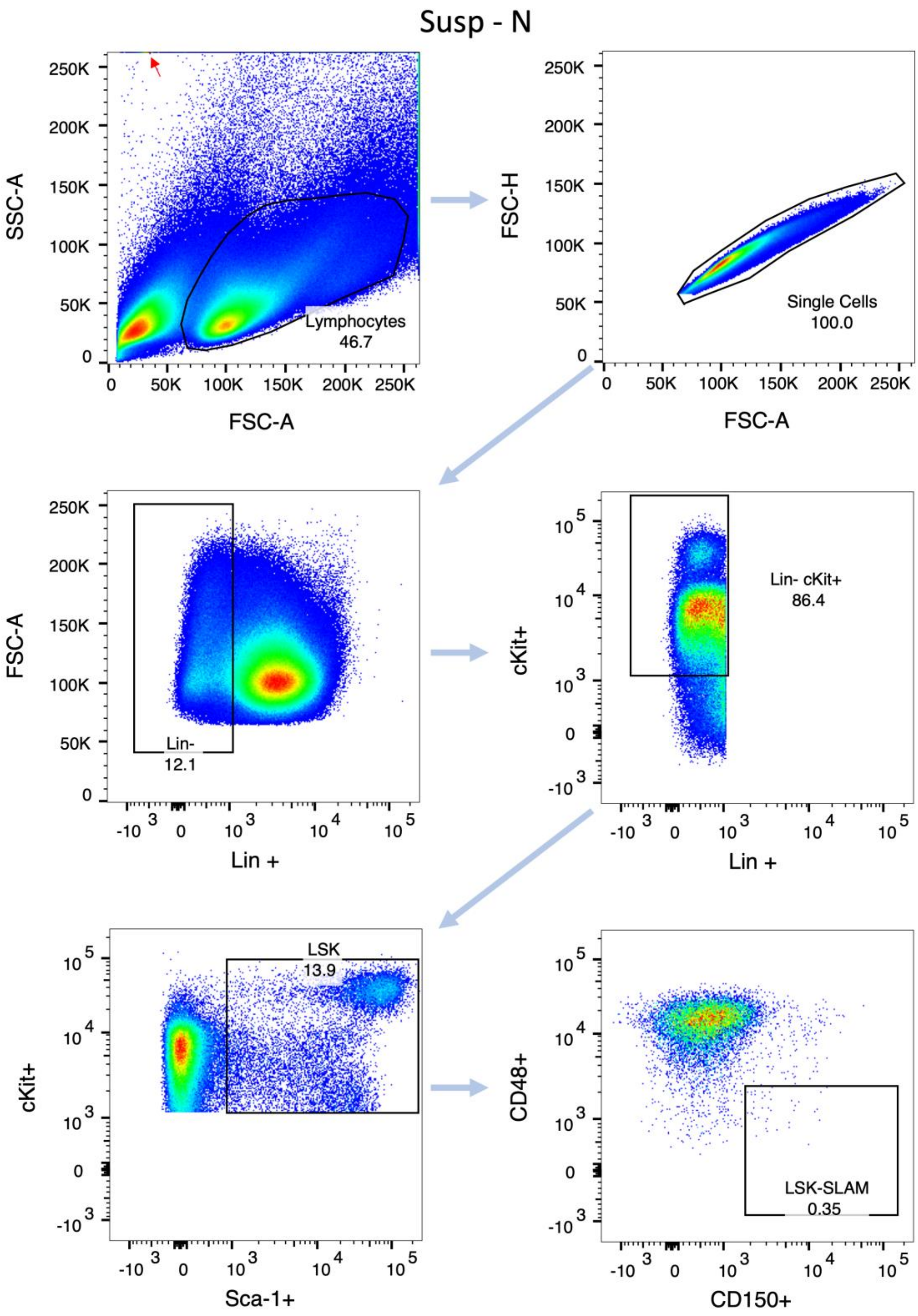

Figure S3f. Representative flow cytometry plots used to identify and quantify the hematopoietic stem/progenitor cell (HSPC) phenotype expression after 10 days of culture starting from Lin-/cKit+ cells in suspension culture (Susp) under normoxia (N). Small red arrows in the FSC-SSC plots denote the position of the counting beads. Debris and dead cells were excluded from the analysis by gating FSC-A/SSC-A. For a better discrimination, double gating with FSC-W/FSC-H and SSC-W/SSC-H was used (not shown).

© 2020 by the authors under the terms and conditions of the Creative Commons Attribution (CC BY) license (http://creativecommons.org/licenses/by/4.0/).
